# Molecular typing of Argentinian *Mycobacterium avium* subsp. *paratuberculosis* isolates by multiple-locus variable number-tandem repeat analysis

**DOI:** 10.1590/S1517-838246220140283

**Published:** 2015-06-01

**Authors:** Andrea Gioffré, Magnolia Correa Muñoz, María F. Alvarado Pinedo, Roberto Vaca, Claudia Morsella, María Andrea Fiorentino, Fernando Paolicchi, Paula Ruybal, Martín Zumárraga, Gabriel E. Travería, María Isabel Romano

**Affiliations:** 1 Instituto Nacional de Tecnología Agropecuaria, Instituto de Biotecnología, Centro de Investigación en Ciencias Veterinarias y Agronómicas, Instituto Nacional de Tecnología Agropecuaria, Buenos Aires, Argentina, Instituto de Biotecnología, Centro de Investigación en Ciencias Veterinarias y Agronómicas, Instituto Nacional de Tecnología Agropecuaria, Buenos Aires, Argentina.; 2 Universidad Autónoma de Baja California, Universidad Autónoma de Baja California, Mexicali, México, Universidad Autónoma de Baja California, Mexicali, México.; 3 Universidad Nacional de La Plata, Centro de Diagnóstico e Investigaciones Veterinarias, Faculdad de Ciencias Veterinarias, Universidad Nacional de La Plata, Chascomús, Argentina, Centro de Diagnóstico e Investigaciones Veterinarias, Faculdad de Ciencias Veterinarias, Universidad Nacional de La Plata, Chascomús, Argentina.; 4 Universidad Nacional de La Plata, Cátedra de Zootecnia Especial II, Faculdad de Ciencias Veterinarias, Universidad Nacional de La Plata, Buenos Aires, Argentina, Cátedra de Zootecnia Especial II, Faculdad de Ciencias Veterinarias, Universidad Nacional de La Plata, Buenos Aires, Argentina.; 5 Instituto Nacional de Tecnología Agropecuaria, Laboratorio de Bacteriología, Estación Experimental Agropecuaria, Instituto Nacional de Tecnología Agropecuaria, Balcarce, Argentina, Laboratorio de Bacteriología, Estación Experimental Agropecuaria, Instituto Nacional de Tecnología Agropecuaria, Balcarce, Argentina.

**Keywords:** paratuberculosis, molecular typing, MIRU-VNTR, MLVA

## Abstract

Multiple-locus variable number-tandem repeat analysis (MLVA) of *Mycobacterium avium* subspecies *paratuberculosis* (MAP) isolates may contribute to the knowledge of strain diversity in Argentina. Although the diversity of MAP has been previously investigated in Argentina using IS*900*-RFLP, a small number of isolates were employed, and a low discriminative power was reached. The aim of the present study was to test the genetic diversity among MAP isolates using an MLVA approach based on 8 repetitive loci. We studied 97 isolates from cattle, goat and sheep and could describe 7 different patterns: INMV1, INMV2, INMV11, INMV13, INMV16, INMV33 and one incomplete pattern. INMV1 and INMV2 were the most frequent patterns, grouping 76.3% of the isolates. We were also able to demonstrate the coexistence of genotypes in herds and co-infection at the organism level. This study shows that all the patterns described are common to those described in Europe, suggesting an epidemiological link between the continents.

## Introduction

Johne's disease or paratuberculosis (PTB) is an enteric disease aetiologically associated with *Mycobacterium avium* subsp. *paratuberculosis* (MAP). This chronic and progressive intestinal infection causes important economic losses to dairy and beef herds around the world (OIE, 2009). Moreover, its zoonotic potential, an issue currently under discussion, is also considered a major concern. MAP has been largely described as restricted to ruminants, such as goats, sheep, cattle, deer, and bison, and recently in camelids (Buergelt *et al.*, 2000; Djønne *et al.*, 2005; Salgado *et al.*, 2009; Attili *et al.*, 2011; Ghosh *et al.*, 2012). Despite this, the isolation of MAP from non-ruminant wildlife, in some cases with pathological lesions, may constitute supporting evidence of a widening of the host range and reservoirs (Beard *et al.*, 2001; Stevenson *et al.*, 2009). Their long-term survival in soil, grass, water samples and biofilms (Whittington *et al.*, 2004; Pickup *et al.*, 2005, Cook *et al.*, 2010), replication and persistence in amoeba (Mura *et al.*, 2006) constitute important evidence that the environment (natural or artificial) may be a major source of MAP strains. These factors increase the exposure of animals and humans to this pathogen and could contribute to the endemicity of PTB. Although epidemiological data are scarce in Argentina, PTB has been well characterised in Buenos Aires province, one of the most productive regions of the country, where a seroprevalence ranging from 7.2 to 19.6% in cattle herds has been reported in the Salado river basin region (INTA-Balcarce, data not published). Additionally, the disease has been widely described in different hosts such as sheep (Nader and Bernardelli, 1986; Jorge *et al.*, 2000; Bernardelli *et al.*, 2002), cattle (Moreira *et al.*, 1999; Paolicchi *et al.*, 2003), captive deer (Paolicchi *et al.*, 2001; Verna *et al.*, 2002), a captive mouflon (*Ovis musimon*) (Bernardelli *et al.*, 2005) and goats (Fiorentino *et al.*, 2012). Several factors such as limitations in diagnostic availability all over the country, the absence of an official infection control programme and the intensification of livestock production systems lead to speculation that PTB will be more widespread in the country in the future. In contrast, bovine tuberculosis (bTB) is better characterised at the epidemiological level in our country. However, although the voluntary official control programme has been successfully applied since 1999, the region is not considered bTB-free. As a consequence, co-infection with *M. bovis* and MAP is plausible in Argentinean herds and flocks (Jorge *et al.*, 2000). Furthermore, PTB also appears to be a possible interference in bTB diagnosis, which has not been estimated in our country. A previous study in the late 1990s performed in bovine and farmed deer isolates from our country suggested a low epidemiological association with Europe. In that study, Moreira and co-workers typed 61 MAP isolates from cattle and deer by IS*900*-restriction fragment polymorphisms analysis (RFLP), and four main patterns were found. The pattern named A was identical to a less frequent pattern, R9 (C17), from Europe, whereas the rest were not found on the same continent (Moreira *et al.*, 1999). Molecular typing based on MIRU (Mycobacterial interspersed repetitive unit) and VNTR (variable number in tandem repeat) loci has been employed as a simple and rapid procedure for differentiation between MAP and other *M. avium* complex isolates (Bull *et al.*, 2003; Radomski *et al.*, 2010). PCR-based genotyping is rapid and inexpensive, and its discriminative power is similar to that previously reported for other techniques such as RFLP (Motiwala *et al.*, 2006; Thibault *et al.*, 2007). Moreover, it has the potential to be applied directly to clinical samples and does not require highly specialised personnel or sophisticated equipment. Consequently, this method is particularly desirable for MAP because of the very slow-growing nature of these organisms (Bull *et al.*, 2003) and could be easily applied in low-income countries. In this study, we aimed to study MAP strain diversity in Argentina by means of MIRU-VNTR.

## Materials and Methods

### MAP isolates and DNA extraction

**Figure 1:**
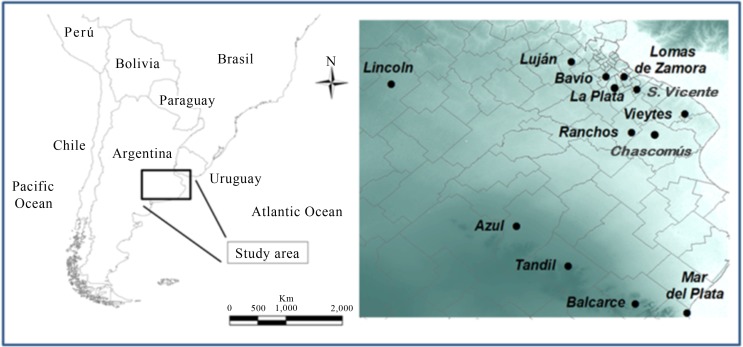
A geographic reference map providing basic geo-information of the study area.

We tested ninety-seven isolates from a MAP collection, as confirmed by their mycobactin J dependence for growth, IS*900*-PCR (Englund *et al.*, 1999) and F57-PCR (Vansnick *et al.*, 2004). These isolates were classified by PCR and restriction endonuclease analysis (PCR-REA) as "cattle type" (C type) (Marsh *et al.*, 1999). The tested clinical isolates corresponded to cattle, (n = 78) goats (n = 16) and sheep (n = 3) belonging to herds (n = 23) from thirteen geographic localities of the Buenos Aires province (Fig. 1). The isolates were obtained over the period from 2005 to 2010. A loopful of each isolate was suspended in sterile distilled water, and cell lysis was performed by serial freeze-boiling cycles. The samples were centrifuged (10,000 × *g*, 5 min), and 2 μL of the supernatant was used as the template in PCR reactions.

### MIRU-VNTR typing: PCR conditions and number of tandem repeat determination

MIRU-VNTR typing was used for eight different MAP-specific markers, as described by Thibault *et al*., and designated VNTR292, MIRUX3 (alias MIRU2 and MIRU3, respectively, Bull *et al.*, 2003; Thibault *et al.*, 2007) VNTR25, VNTR47, VNTR3, VNTR7, VNTR10 and VNTR32 (Thibault *et al.*, 2007). The primers and PCR conditions were as suggested previously (Thibault *et al.*, 2007), with minor modifications*.* The amplification mixture consisted of 1X buffer (10 mM Tris-HCl pH 9; 50 mM KCl and 0.1% Triton X-100, 2.5 mM MgCl_2_) 0.2 mM each dNTP, 1 μM each primer and 1.25 U GoTaq polymerase (Promega). MgCl_2_ was supplemented for MIRUX3 (2 μL/reaction), and DMSO and betaine were supplemented when amplifying VNTR loci 47, 3, 7, 10 and 32, as suggested previously. The described previously annealing temperatures were employed, except for locus 47, for which the annealing temperature during the first 10 cycles was decreased 1 °C per cycle from 69 °C to 59 °C, according to a touch-down protocol, and then a final annealing temperature at 64 °C was selected for 35 cycles. The PCR products were resolved by 3.5% agarose gel electrophoresis, and the molecular weights were determined using 100 bp and 50 bp DNA markers (Promega Corp.) and BioNumerics software (Applied Maths, Belgium). The number of tandem repeats in each locus was assigned according to a previous report (Radomski *et al.*, 2010; see Supplementary tables). Numerical profiles were classified according to INMV (INRA, Nouzilly, MIRU-VNTR) combinations, as suggested previously (Thibault *et al.*, 2007; Radomski *et al.*, 2010). DNA from *M. avium* subsp. *paratuberculosis* K10 was included as a control (INMV2).

### Index of discrimination

The allelic diversity for each locus and the discriminatory power of complete MIRU-VNTR scheme typing were determined using the Hunter and Gaston discriminatory index (Hunter, 1990; Hunter and Gaston, 1988) calculated using Simpson's index of diversity formula, as follows:

$$D=1-\frac{1}{N(N-1)}\sum_{j=1}^{s}x_{j}(x_{j}-1)$$

where *D* corresponds to the index of discriminatory power, *N* to the number of unrelated strains tested, *S* to the number of different types, and *xj* to the number of strains belonging to the jth type, assuming that strains will be classified into mutually exclusive categories.

This index was estimated considering epidemiologically unrelated strains and was calculated using the on-line software: http://insilico.ehu.es/mini_tools/discriminatory_power/, University of the Basque Country.

### Epidemiological study

The eBURST algorithm described by Feil *et al.* (2004) was initially developed for MLST but is also suitable for MLVA studies. The goe-BURST algorithm (goeburst.phyloviz.net/) (Francisco *et al.*, 2009), which uses the same clustering rules as eBURST (Feil *et al.*, 2004) but provides a global optimal solution, was used to determine relationships between the obtained profiles. Clonal complexes were defined as MIRU-VNTR loci that are linked through single-locus variants (SLVs) and named on the basis of the predicted founder, which is the MIRU-VNTR pattern associated with most SLVs.

## Results

### MIRU-VNTR genotypes: Frequency, hosts and geographic distribution

**Figure 2:**
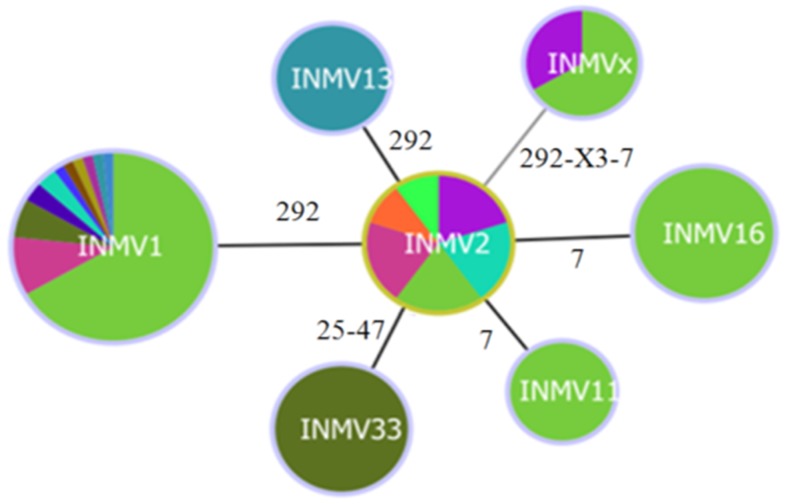
GoeBURST clustering of the seven MIRU-VNTR profiles. Locations are represented in different colours. Differential loci between the patterns are shown in black numbers. The size of the pie is related to the number of samples.

The overall analysis yielded seven different INMV MIRU-VNTR patterns in the MAP isolates studied (n = 97). The INMV1 pattern grouped 65% of the isolates and was the most frequent. The rest of the genotypes were less represented, with INMV2 (11.3%), INMV16 (8.2%), and INMV33 (7.2%) being almost equally frequent. Although we were not able to amplify loci 292 and 7 in three isolates, the other loci indicated a pattern different from those mentioned above. This incomplete pattern represented 3.1% of the isolates. The most frequent patterns (INMV1 and INMV2) were described in at least two hosts (cattle, goat and sheep or cattle and goat, respectively), whereas the less frequent ones (INMV 11, INMV13, INMV16, INMV33, INMVx) were related to cattle (Table 1). The most common MIRU-VNTR patterns were widely distributed geographically, which limits their usefulness for tracing the geographic spread of infection. The MAP isolates from San Vicente showed the pattern INMV 13, and this pattern occurs exclusively in that location. The patterns INMV 11, INMV 16 and INMV 33 were exclusively from Tandil (Table 1 and Fig. 2).

**Table 1:** MIRU-VNTR patterns, host and geographical distribution.

MIRU-VNTR pattern^1^	TRs at MIRU-VNTR locus^2^								No. of isolates (%)	Host^3^	Geographical origin^4^
				
	292	X3	25	47	3	7	10	32			
INMV 1	4	2	3	3	2	2	2	8	63 (65)	C, G, S	T, SV, Ch, V, R, A, LP, Li, MP, Ba
INMV 2	3	2	3	3	2	2	2	8	11 (11.3)	C, G	T, LZ, Lu, LP, B, Li
INMV 11	3	2	3	3	2	4	2	8	2 (2.1)	C	T
INMV 13	2	2	3	3	2	2	2	8	3 (3.1)	C	SV
INMV 16	3	2	3	3	2	5	2	8	8 (8.2)	C	T
INMV 33	3	2	5	2	2	2	2	8	7 (7.2)	C	T
INMVx^5^	N	1	3	3	2	N	2	8	3 (3.1)	C	T, LZ

1 Designated according to the INRA Nouzilly MIRU-VNTR patterns (Thibault *et al.*, 2007).

2 Loci were arranged in the same order as that of Thibault *et al.* (2007). TRs: tandem repeats.

3 Host: C, cattle; G, goats; S, sheep.

4 Letter locality code: T, Tandil; SV, San Vicente; Ch, Chascomús; V, Vieytes; R, Ranchos; A, Azul; LP, La Plata; Li, Lincoln; LZ, Lomas de Zamora; Lu, Luján; B, Balcarce; MP, Mar del Plata; Ba, Bavio.

5 non-determined pattern; N denotes no amplification of the locus.

### Analysis of profiles by individual herds

Those herds with more than one isolate were considered for studying the coexistence of different genotypes. We found more than one strain in five out of nine herds. The herd with the highest number of isolates studied (Herd I, Tandil, n = 39) showed the highest diversity (five patterns) (Table 2). Moreover, two isolates were analysed from one animal of this herd, and two patterns were confirmed (not shown). The patterns found (INMV1 and INMV16) were the most frequent in this herd.

**Table 2:** Analysis of diversity by herd.

Herd^1^ no. (district)	MIRU-VNTR pattern	TRs at loci: 292-X3-25-47-3-7-10-32	No. of isolates/genotype	Host
I (Tandil)	INMV1	42332428	25	cattle
	INMV16	32332528	7	
	INMV11	32332428	2	
	INMV2	32332228	3	
	INMVx	N1332N28	2	
II (San Vicente)	INMV13	22332228	3	cattle
	INMV1	42332428	1	
III (L. de Zamora)	INMV2	32332228	2	cattle
	INMVx	N1332N28	1	
IV (Chascomús)	INMV1	42332428	2	cattle
V (Lincoln)	INMV33	32522228	10	cattle
	INMV1	42332428	5	
VI (Mar del Plata)	INMV1	42332428	15	goat
VII (La Plata)	INMV1	42332428	2	sheep
VIII (La Plata)	INMV1	42332428	1	cattle
	INMV2	32332228	1	
IX (Balcarce)	INMV1	42332428	2	cattle

1 Only herds represented by more than one isolate were considered.

2 Designated according to the INRA Nouzilly MIRU-VNTR patterns (Thibault *et al.*, 2007).

3 Loci were arranged in the same order as that of Thibault *et al.* (2007). TRs: Tandem repeats.

### Allelic diversity and discriminatory power

Thirty-two non-epidemiologically related isolates were resampled to carry out both allelic diversity and discriminatory power analyses. According to our study, VNTR292 showed the maximum allelic diversity for Argentinean MAP strains (0.521). In contrast, VNTR loci 3, 10 and 32 were un-informative. The discriminatory power of the remaining MIRU-VNTR loci (X3, 25, 47 and 7) was weak, similar to most previous studies (Table 3). With the exception of VNTR32, which has eight tandem repeats (TRs), the rest of the loci showed a TR copy number lower than five. MIRU X3 was the only locus with one tandem repeat, and alleles without TRs were absent in the studied isolates. The complete MIRU-VNTR scheme discriminatory index D reached 0.6331. However, the discriminatory index was unchanged when the number of MIRU-VNTR loci was reduced to VNTR292-MIRUX3-VNTR25-VNTR47 and VNTR7, and the exclusion of VNTR47 or VNTR25 from the last combination did not reflect a significant difference in the discriminatory power reached (0.6086). Therefore, these findings indicate that a reduced MIRU-VNTR scheme (VNTR292-MIRUX3-VNTRs25/47 and VNTR7) yields discriminatory values comparable to the scheme of the complete 8 loci.

**Table 3:** Frequency of tandem repeats in each MIRU-VNTR locus and comparative allelic diversity between different studies.

MIRU-VNTR locus	No. of isolates with TR copy no:										Allelic diversity^1^				
	
	0	1	2	3	4	5	6	7	8	9	This study	Study I	Study II	Study III^2^	Study IV
292^3^			1	11	18						0.521	0.595	0.58	0.561	0.51
X3^3^		2	30								0.121	0.342	0.09	0.11	0.04
25				31		1					0.062	0.394	0.1	0.209	0.07
47			1	31							0.062	0.269	0.06	-	0.05
3			32								0	0.077	0.2	-	0.005
7			28		1	1					0.131	0.480	0.22	-	0.19
10			32								0	-	0.24	-	0.18
32									32		0	0.077	0.006	0.118	0.064

1 Allelic diversity compared to those previously reported by Möbius *et al.*, 2008 (Study I), Stevenson *et al.*, 2009 (Study II), Castellanos *et al.*, 2010 (Study III) and Radomski *et al.*, 2010 (Study IV).

2 The reported allelic diversity from type II strains was selected.

3 VNTR292 and MIRUX3 are referred to as MIRU2 and MIRU3, respectively, in Study I and Study III, as defined by Bull *et al.* (2003).

### goeBURST analysis

To study the relationship among MIRU-VNTR patterns, we performed a cluster analysis using the goeBURST algorithm. The goeBURST clustering suggested pattern INMV2 as the primary founder. This pattern, together with INMV1, was the most geographically widespread, and only these two patterns were isolated from different hosts (Figure 2 and Table 1).

## Discussion

The import of live animals from the United Kingdom is considered the most probable route of the introduction of *M. bovis* to Argentina, and this fact is supported by the genotyping of isolates from both regions (Cataldi *et al.*, 2002; Smith *et al.*, 2011). Considering the nature of MAP, we hypothesised that the genotypes/patterns of MAP from our country and Europe are also common. Our results expand an earlier study that examined the diversity of Argentinean MAP isolates using IS*900*-RFLP (Moreira *et al.*, 1999). MIRU-VNTR is faster and less labour-intensive than RFLP and has been internationally adopted (Stevenson *et al.*, 2009, Inagaki *et al.*, 2009, Castellanos *et al.*, 2010; Radomski *et al.*, 2010; Fernández-Silva *et al.*, 2011, Biet *et al.*, 2012). Hence, we selected a previously reported MIRU-VNTR scheme to study the diversity of circulating strains in Argentina in an effort to eventually perform a comparative analysis between different locations. We studied a larger sample size from three different hosts, covering a broad area of study compared to the aforementioned study. We demonstrate here that the most frequent MIRU-VNTR pattern is INMV1 (65%). This result is concordant with a previous report in which 51% of European isolates were typed as INMV1, which was also shown to be distributed in 6 out of the 7 countries assessed in that study (Stevenson *et al.*, 2009). Additionally, patterns INMV1 and INMV2 were found to represent most of the isolates (36% and 34%, respectively) from 10 different countries and hosts (Thibault *et al.*, 2007). The goeBurst analysis indicated INMV2 as the founder genotype in our study. A similar result was obtained by the minimum-spanning-tree approach performed in a strain panel containing mainly European isolates (Radomski *et al.*, 2010).

To date, the patterns INMV1, INMV2, INMV11, INMV13, INMV16, INMV33 (this work), and INMV8 (Thibault *et al.*, 2007) have been described in Argentinean isolates. The currently available information for pattern distribution in the United Kingdom shows the presence of INMV1, INMV2, INMV19, INMV21, INMV25 (Stevenson *et al.*, 2009) and INMV14 and INMV33 (Thibault *et al.*, 2007) in different hosts. Considering these data in combination with the fact that INMV1 and INMV2 are distributed worldwide, we cannot assume or discard the epidemiological link between the UK and Argentina using this VNTR-MIRU typing approach, and further evidence is required to confirm this hypothesis. Nonetheless, almost all described patterns in our study have been previously reported in European isolates (Thibault *et al.*, 2007). The combination of other typing technique(s) with MIRU-VNTR analysis has enabled an improvement of the discriminatory power (Thibault *et al.*, 2008; Stevenson *et al.*, 2009). More recently, Fernández Silva *et al*. were able to improve the discrimination between six INMV1 Argentinian strains, which were resolved into three genotypes using multilocus short sequence repeats (Fernández Silva *et al.*, 2012). Hence, the use of more than one typing method to further discriminate between isolates and the inclusion of a larger number of strains by random sampling could be more informative about the real epidemiological scenario in Argentina.

We found that the most important limitation of MIRU-VNTR typing was the lack of amplification for two loci. We herein report INMVx as an incomplete pattern because two MIRU-VNTR loci (292 and 7) remained refractory to amplification in three samples. This could be due to target sequence polymorphisms or to the absence of these loci in these strains.

The discriminatory index we obtained reached 0.63, in accordance with previous studies by Thibault *et al.* (2007) and Stevenson *et al.* (2009), 0.75 and 0.64, respectively. Based on information derived from these previous studies and the present work, a reduced scheme can be considered for typing MAP isolates without a significant loss of discriminative power. In this regard, MIRU-VNTR loci 3, 10 and 32 can be excluded in future studies involving local strains, and moreover novel markers could be considered for improving the discriminatory power.

The coexistence of different genotypes within cattle herds has been previously reported (van Hulzen *et al.*, 2011; Fernández-Silva *et al.*, 2011). Within this context, cattle herd I (Tandil) with at least five different patterns constitutes a good example. The observed genotype co-existence within herds strongly suggests that a lack of animal monitoring prior to their introduction into a herd may represent a factor that is key to the observed diversity. Accordingly, we cannot rule out the presence of more than one genotype in all the tested herds, and a wider sampling should be performed to examine this. For most of the herds assessed in this study, animals are currently introduced, or were introduced in the past, without prior testing, justifying this practice based on the apparent healthy condition of the animals. One case was the introduction of a bull for breeding purposes in an infected herd (herd II, San Vicente). The isolate from this animal showed a pattern different from those described for other isolates from the same herd. Considering that animals usually become infected early in their life, one can assume that this bull was already infected. However, we also studied a MAP-infected flock (herd VI, Mar del Plata) in which the PTB seroprevalence was 48% (Fiorentino *et al.*, 2012). In this case, the introduction of foreign animals was not a common practice, which clearly correlates with the presence of isolates sharing the same pattern. It is important to mention that, unfortunately, this MAP-infected herd is a frequent supplier of animals to neighbouring herds.

Since the early epidemiological studies on PTB, molecular typing techniques have been described and applied. The MIRU-VNTR approach has been used to type *M. avium* complex strains from different countries, such as France, Germany, Czech Republic, Finland, Greece, The Netherlands, Norway, Scotland, Spain, Japan and more recently Colombia and Argentina (Möbius *et al.*, 2008; Stevenson *et al.*, 2009; Inagaki *et al.*, 2009; Castellanos *et al.*, 2010; Radomski *et al.*, 2010; Fernández-Silva *et al.*, 2011, this work). Despite the high acceptance of this technique, the results from different studies are often difficult to compare due to the lack of standardisation. At present, a consensus on which is the best technique or combination of multiple genotyping techniques is urgently needed to definitively understand the global epidemiology of PTB, and this can be achieved through collaborative work. This report constitutes the first MIRU-VNTR study of a large panel of strains from one of the most productive regions of Argentina.
